# Rabies vaccine stockpile: fixing the supply chain

**DOI:** 10.2471/BLT.16.183012

**Published:** 2016-09-01

**Authors:** Bernadette Abela-Ridder, Stephen Martin, Gyanendra Gongal, Dirk Engels

**Affiliations:** ^a^ Department of the Control of Neglected Tropical Diseases, World Health Organization, avenue Appia 20, 1211 Geneva 27, Switzerland.; b Department of Pandemic and Epidemic Diseases, World Health Organization, Geneva, Switzerland.; c Department of Health Security and Emergency Response, WHO Regional Office for South-East Asia, New Delhi, India.

World Rabies Day is 28 September, 2016,^1^ and is designed to raise awareness about the prevention and control of this neglected disease. Almost all human rabies are transmitted by domestic dog bites or scratches, usually via saliva. Rabies virus replicates in the wound site and gains access to nerves to reach the central nervous system. The incubation period varies from five days to several years. By the time of clinical onset, the virus is widely disseminated throughout the central nervous system and the infection is invariably fatal. Prevention of human rabies and control of canine rabies have been successful in north America, western Europe and a number of Asian and Latin American countries through vaccination of dogs, responsible dog ownership, enforcement of leash laws, and provision of life-saving bite treatment. Pre-exposure immunization is strongly recommended for people in high-risk occupations such as laboratory workers dealing with live rabies virus, vaccinators and people involved in any activity that might bring them professionally or otherwise into direct contact with bats, carnivores and other mammals in rabies-affected areas.

The World Health Organization (WHO), the World Organisation for Animal Health (OIE), the Food and Agriculture Organization of the United Nations (FAO) and the Global Alliance for Rabies Control (GARC) have committed to eliminating rabies deaths in humans by 2030.^2^ As Margaret Chan, WHO’s Director-General, said: “Rabies belongs in the history books.”

As for many neglected diseases, data are suboptimal. An estimated 59 000^3^^,^^4^ people die from rabies every year, despite the existence of effective vaccines. Around 90% of these deaths occur among children living in rural areas in Africa and Asia,^5^ almost all as a consequence of dog bites.^6^

The world has many competing disease-control priorities, and rabies has fallen off the global health agenda. Rabies control requires two complementary interventions. Mass dog vaccination programmes are needed to break dog-to-human transmission and people who are exposed to rabies need prompt and effective treatment. Such treatment includes wound care, immunoglobulin and vaccination. WHO, with its partners and stakeholders, are quantifying the resources required to implement these programmes on a scale sufficient to end human rabies deaths. 

WHO’s current rabies vaccine position paper^7^ states that four to five courses of the vaccine must be given with rabies immunoglobulin to all people who have sustained bites that perforate the skin. Immunoglobulins provide passive immunity until the vaccine has stimulated the immune system. However, rabies immunoglobulins are expensive. One vial is about 39 United States dollars, and two or more vials are usually needed.^8^ Immunoglobulins have to be maintained at 2–8 °C, and are difficult to procure in most countries. Because it is a biological product, rabies immunoglobulin is not covered by WHO’s prequalification procedures. Currently four vaccines are pre-qualified by WHO.^9^

WHO’s Strategic Advisory Group of Experts on Immunization has initiated a review of its rabies position paper. This group will review the use and scheduling of rabies vaccines and immunoglobulins in view of scientific evidence, programmatic feasibility and clinical practice in countries with a high incidence of dog bites. The group will also assess evidence for new vaccines and for those vaccines in the process of obtaining WHO prequalification or national market authorization.

Human rabies vaccine is not included in the routine vaccines covered by the expanded programme on immunization. Many countries therefore have difficulty measuring and forecasting demand for rabies vaccine. A lack of good data causes procurement delays and stock shortages. In desperation, countries may source vaccines from manufacturers that do not have WHO prequalification, buying vaccines at inflated prices and without the quality assurance that prequalification brings.

To provide a reliable source of vaccines for countries facing these difficulties in procurement, WHO is planning to create a human rabies vaccine stockpile to match the dog rabies vaccine bank established by OIE.^10^ By the end of 2017, countries will be able to rapidly obtain quality-assured vaccines. As has happened with other vaccine stockpiles,^11^ this mechanism will generate a demand and supply cycle that can be reliably quantified. By drawing on WHO’s stockpile, countries will contribute to stabilizing demand for manufacturers. In aggregating global requirements, WHO will be able to broker reliable supplies and assist countries in forecasting their needs.

The world has all the tools needed to prevent human deaths from rabies. These tools need to be on hand where and when people are exposed. The risk of contracting rabies from animal bites needs to be reduced by animal vaccination and bite prevention programmes. Vaccines, immunoglobulins and wound care have to be provided in all settings where people are exposed. The animal and human health sectors must coordinate efforts and improve community awareness and engagement. More money and political commitment will prevent deaths from this zoonotic disease. WHO and its partners are working to improve the evidence, update technical guidance and create a vaccine stockpile that will help countries reach the global target of preventing human deaths from rabies.
