# Corrigendum

**DOI:** 10.2471/BLT.16.111016

**Published:** 2016-10-01

In Volume 94, Issue 9, September 2016, page 635, the third sentence of the third paragraph should read: "Around 40% of these deaths occur among children living in rural areas in Africa and Asia,^5^ almost all as a consequence of dog bites.^6^"

